# The utility of the Japanese Association for Acute Medicine DIC scoring system for predicting survival in acute exacerbation of fibrosing idiopathic interstitial pneumonia

**DOI:** 10.1371/journal.pone.0212810

**Published:** 2019-08-19

**Authors:** Ryo Yamazaki, Osamu Nishiyama, Sho Saeki, Hiroyuki Sano, Takashi Iwanaga, Yuji Tohda

**Affiliations:** Department of Respiratory Medicine and Allergology, Kindai University, Faculty of Medicine, Osakasayama, Osaka, Japan; Toranomon Hospital, JAPAN

## Abstract

**Background:**

Although evidence of a disseminated intravascular coagulation (DIC)-like reaction has been identified in the lung parenchyma of patients with acute exacerbation of idiopathic pulmonary fibrosis (IPF), an association between DIC and IPF outcome has not been elucidated. Therefore, we retrospectively investigated the association between the Japanese Association for Acute Medicine (JAAM)-DIC score and mortality in patients with acute exacerbation of fibrosing idiopathic interstitial pneumonia (AE-fIIP).

**Methods:**

Between January 2008 and December 2016, consecutive patients with chronic fIIP who were admitted for the first time for AE-fIIP were recruited into the study. Associations between clinical data and JAAM-DIC score at the time of admission and mortality were examined.

**Results:**

During the study period, a total of 91 patients with fIIP (73.0±8.4 y.o.) were hospitalized for AE-fIIP for the first time. The 30-day and hospital mortality were 8.7% and 17.5%, respectively. A multivariate analysis showed that the JAAM-DIC score on admission was an independent predictor of 30-day mortality (odds ratio [OR] 2.57, 95% confidential interval [CI] 1.50–4.40, *P* = 0.0006). The APACHE II score (OR 1.29, 95% CI 1.01–1.63, *P* = 0.03) and the JAAM-DIC score (OR 3.47, 95% CI 1.73–6.94, *P* = 0.0004) were independent predictors of hospital mortality.

**Conclusions:**

The JAAM-DIC scoring system can predict survival in patients with AE-fIIP. The role of DIC in the pathogenesis of AE-fIIP merits further investigation.

## Introduction

Idiopathic pulmonary fibrosis (IPF) is the most common type of idiopathic interstitial pneumonia (IIP). IPF is chronic and progressive; the prognosis of patients with IPF is generally poor and the median survival time from diagnosis is 2 to 3 years [1, 2]. However, the clinical course of IPF is highly variable. Some patients progress slowly, some show relatively rapid deterioration, and some have acute exacerbations. Acute exacerbation of IPF (AE-IPF) is defined as a rapid worsening of respiratory symptoms associated with new ground-glass opacities or consolidations of unknown etiology, and is associated with high mortality [1]. The incidence of AE-IPF varies among studies, but it is estimated to occur in 5%-10% of IPF patients annually [1]. Furthermore, AE-IPF decreases survival in patients with IPF.

Disseminated intravascular coagulation (DIC) is a condition characterized by the systemic intravascular activation of blood coagulation. Intravascular fibrin clots are formed, leading to the thrombotic obstruction of small- and midsized vessels. Furthermore, DIC can result in compromised blood supplies to organs and can contribute to multiple organ failure [3]. DIC has been well validated as an independent predictor of survival, doubling the risk of mortality in patients with severe sepsis and trauma [4].

It was previously reported that disordered coagulation, fibrinolysis, and endothelial damage were found in autopsy samples of patients with AE-IPF [5]. An increase in coagulation markers such as thrombomodulin in bronchoalveolar lavage (BAL) fluid from patients with AE-IPF was also reported [6]. Therefore, it is reasonable to speculate that pulmonary DIC, or a DIC-like reaction, may play a role in AE-IPF.

DIC can be difficult to diagnose. Recently the Japanese Association for Acute Medicine (JAAM) developed a scoring system for diagnosing DIC that is simple and easy to use [7], and is widely used in Japan. We hypothesized that the JAAM-DIC score may predict mortality in patients with AE-IPF and/or other types of fibrosing IIP (fIIP).

Taking into account that acute exacerbations can also occur in idiopathic non-specific interstitial pneumonia (NSIP) [8], we retrospectively investigated the association of DIC score with mortality in patients with acute exacerbation of fIIP (AE-fIIP) to test the hypothesis that DIC, identified according to the JAAM-DIC score, can predict survival in patients with AE-fIIP.

## Methods

### Patients

Between January 2008 and December 2016, consecutive patients with chronic fIIP who had their first admission for AE-fIIP to the Kindai University Hospital were recruited into the study. The diagnosis of IPF was made according to the recent official statement [1]. Chronic fIIPs other than IPF were defined using the following criteria: 1) exclusion of other known causes of interstitial lung disease such as domestic and occupational environmental exposures, connective tissue disease, and drug toxicity; 2) the presence of possible usual interstitial pneumonia (UIP) pattern or inconsistent with UIP pattern on high-resolution computed tomography (HRCT) in patients not subjected to surgical lung biopsy (SLB); and 3) pathologically diagnosed idiopathic UIP or NSIP in patients subjected to SLB [9]. Acute exacerbation of chronic fIIP was defined using a modification of a recent international working group report as follows: 1) acute worsening or development of dyspnea of less than one month duration; 2) HRCT with new bilateral ground-glass opacities and/or consolidation superimposed on a background of interstitial pneumonia; 3) deterioration not fully explained by cardiac failure or fluid overload [10].

Informed consent was waived because this study was based on a retrospective analysis of case records from our university hospital. Approval for the use of these data and the analysis was provided by the ethics committee of the Kindai University, Faculty of Medicine (No. 30–044).

### Pulmonary function tests

The most recent pulmonary function tests, performed within 1 year prior to acute exacerbation, were used to assess baseline pulmonary function. Pulmonary function tests were performed (CHESTAC-8800; Chest, Tokyo, Japan) according to the standards proposed by the European Respiratory Society [11, 12]. The assessments included spirometry and single-breath measurements of diffusing capacity for carbon monoxide (DLco). Results are expressed as absolute values and as percentages of Japanese normal predictive values [13, 14].

### Data collection

Routine blood sampling was performed on admission. The DIC score was calculated according to the JAAM scoring system. The scoring system evaluates the following variables [7]: 1) systemic inflammatory response syndrome (SIRS) score [15], 2) platelet count, 3) prothrombin time-international normalized ratio (PT INR), and 4) fibrinogen and fibrin degradation products (FDP). DIC is diagnosed when the total JAAM-DIC score is ≥4 out of possible 8. The JAAM-DIC scoring system has been validated as a useful clinical tool for the early diagnosis of DIC in diverse patient populations [7, 16–19]. The acute physiology and chronic health evaluation II (APACHE II) score and the sequential organ failure assessment (SOFA) score were also calculated [20–22]. The SIRS score includes the following items: 1) body temperature >38°C or <36°C, 2) heart rate >90 beats/min, 3) respiratory rate > 20 breath/min or PaCO_2_ <32 mmHg, and 4) white blood cell count >12,000/μL or <4,000/μL, or >10% immature neutrophils [15].

### Assessment of survival

Survival was evaluated at 30 days. Total hospital mortality was also assessed. All deaths were confirmed by hospital chart review.

### Statistical analysis

Continuous variables were expressed as means±SD and categorical variables were expressed as frequencies. Univariate and multivariate analyses with logistic regression models were used to reveal potential risk factors for 30-day and hospital mortality. Significant variables identified by univariate analysis were further analyzed by stepwise multivariate analysis. A *P* value of <0.05 was considered statistically significant. Analyses were performed with the PASW statistical package version 18 (SPSS Japan Inc., Tokyo, Japan).

## Results

During the study period, 91 patients with fIIP were hospitalized a total of 113 times for AE. The diagnosis of fIIP was made by SLB in 6 patients (IPF/UIP;5, NSIP;1) and the other 85 patients were diagnosed clinically.

Clinical characteristics of the patients are shown in Table 1 (S1 Datasheet). The mean age was 73.0±8.4 years, with 63 men and 28 women. Mean FVC was 72.6±20.9% predicted, and mean DLco was 63.3±21.7% predicted. Other clinical data at the first admission for AE-fIIP are shown in Table 2. The mean values for C reactive protein (CRP) levels, white blood cell count (WBC), platelet counts, and partial pressure of arterial oxygen/fraction of inspiratory oxygen (PaO_2_/FiO_2_) were 6.3±6.9 mg/dL, 9185±3806 /μL, 25.9±9.2 ×10^4^/μL, and 261±88, respectively. The mean JAAM-DIC score was 0.7±1.2. The results of BAL fluid analysis are shown in Table 2, although some patients could not undergo bronchoscopy because of severe respiratory symptoms.

**Table 1:** Characteristics of all study patients with fIIP and a subset with IPF diagnosed at the first acute exacerbation.

Characteristics	All patients n = 91	IPF diagnosed n = 42
Age, yr	73.0 ± 8.4	72.9 ± 5.9
Sex, n (%)		
Male	63 (69.2)	37 (88.0)
Female	28 (30.7)	5 (11.9)
Body mass index	23.4 ± 3.6^a^	22.2 ± 2.8^b^
Pulmonary function tests		
FVC, L	2.1 ± 0.7^c^	2.1 ± 0.8^e^
FVC, % predicted	72.6 ± 20.9^c^	68.7 ± 23.4^e^
FEV_1_, L	1.7 ± 0.6^c^	1.8 ± 0.6^e^
FEV_1_/FVC, %	82.5 ± 9.6^c^	86.7 ± 6.1^e^
DLco, mL/min/mmHg	9.2 ± 3.3^d^	9.7 ± 2.8^f^
DLco, % predicted	63.3 ± 21.7^d^	66.8 ± 15.8^f^
Smoking status, n (%)		
yes/no	63/28 (69.2/30.7)	34/8 (80.9/19.0)
current/former/never	2/61/28 (2.1/67.0/30.7)	1/33/8 (2.3/78.5/19.0)
Treatment of interstitial pneumonia at baseline, n (%)		
Pirfenidone	6 (6.5)	6 (14.2)
Corticosteroid	2 (2.1)	0 (0)
Corticosteroid plus tacrolimus	2 (2.1)	2 (4.7)
Corticosteroid plus cyclosporine	1 (1.0)	0 (0)
Corticosteroid plus cyclophosphamide	1 (1.0)	0 (0)
Corticosteroid, cyclosporine, and Pirfenidone	1 (1.0)	1 (2.3)
None	78 (85.7)	33 (78.5)
Long-term oxygen therapy, n (%)		
yes/no	17/74 (18.6/81.3)	10/32 (23.8/76.1)

DLco = diffusing capacity for carbon monoxide, FEV1 = forced expiratory volume in 1 second, fIIP = fibrosing idiopathic interstitial pneumonia, FVC = forced vital capacity, IPF = idiopathic pulmonary fibrosis.

Values are shown as actual numbers or means±standard deviation.

^a^:n = 86

^b^:n = 38

^c^:n = 65

^d^:n = 41

^e^:n = 27

^f^:n = 17

**Table 2:** Clinical data of all study patients and a subset with IPF diagnosed at the first acute exacerbation.

Characteristics	All patients n = 91	IPF diagnosed n = 42
CRP, mg/dL	6.3 ± 6.9	9.5 ± 6.7
WBC, /μL	9185 ± 3806	11004 ± 4200
LDH, IU/L	329 ± 136	340 ± 114
Platelet counts, ×10^4^/μL	25.9 ± 9.2	28.7 ± 10.6
Fibrinogen, mg/μL	472 ± 178^a^	544 ± 158^f^
FDP, μg/mL	8.9 ± 13.5^b^	11.0 ± 17.8^g^
D-dimer, μg/mL	3.3 ± 5.2^c^	4.2 ± 7.3^h^
KL-6, U/mL	1644 ± 1468	1227 ± 936
PaO_2_ / FiO_2_ ratio	261 ± 88	237 ± 94
SOFA score	2.2 ± 1.4^d^	2.6 ± 1.1^i^
JAAM-DIC score	0.7 ± 1.2^e^	1.0 ± 1.1^g^
APACHE II score	7.7 ± 3.7^d^	10.0 ± 5.0^i^
Duration of hospitalization, days	46.6 ± 48.2	56.3 ± 63.0
BAL findings		
Total cell count, 10^5^/mL	2.8 ± 5.0 ^j^	2.0 ± 1.2 ^k^
Macrophage, %	48.4 ± 22.7 ^j^	45.7 ± 19.9^k^
Lymphocyte, %	31.5 ± 20.7 ^j^	29.2 ± 21.3 ^k^
Neutrophil, %	14.3 ± 17.9 ^j^	19.7 ± 18.6^k^
Eosinophil, %	5.6 ± 6.6 ^j^	4.7 ± 5.5^k^
CD4/CD8	2.8 ± 2.9 ^j^	3.3 ± 2.8^k^

APACHE II = Acute Physiology and Chronic Health Evaluation II, BAL = bronchoalveolar lavage, CD4 = cluster of differentiation 4, CD8 = cluster of differentiation 8, CRP = C-reactive protein, FDP = fibrinogen and fibrin degradation products, IPF = idiopathic pulmonary fibrosis, JAAM-DIC = Japanese Association for Acute Medicine- disseminated intravascular coagulation, KL-6 = Krebs von der Lungen-6, LDH = lactate dehydrogenase, PaO_2_ / FiO_2_ = partial pressure of atrial oxygen / fraction of inspiratory oxygen, SOFA = the sequential organ failure assessment, WBC = white blood cell.

Values are shown as mean±standard deviation.

^a^:n = 74

^b^:n = 79

^c^:n = 72

^d^:n = 82

^e^:n = 81

^f^:n = 36

^g^:n = 40

^h^:n = 38

^i^:n = 37

^j^:n = 63

^k^:n = 25

Treatments for acute exacerbation are shown in Table 3. All 91 patients were treated with corticosteroids, including 74 patients (81.3%) who received corticosteroid pulse therapy (methylprednisolone at 1000 mg/day for 3 days). Twenty-nine patients (31.8%) received treatment with immunosuppressive agents.

**Table 3:** Treatment for acute exacerbations.

Characteristics	All patients n = 91	IPF diagnosed n = 42
Methylprednisolone pulse, N (%)	74 (81.3)	36 (85.7)
Corticosteroid high dose, N (%)	11 (12.1)	5 (11.9)
Corticosteroid low dose, N (%)	6 (6.6)	1 (2.4)
Cyclosporine, N (%)	20 (22.0)	6 (14.3)
Cyclophosphamide, N (%)	9 (9.9)	6 (14.3)
PMX, N (%)	4 (4.4)	4 (9.5)

IPF = idiopathic pulmonary fibrosis, PMX = polymyxin B-immobilized fiber column.

Actual numbers are shown, with percentages in parentheses. High-dose corticosteroid means ≧0.6 mg/kg/day.

The 30-day and hospital mortality rates were 8.7% and 17.5%, respectively. In the subset of patients with diagnosed IPF, the 30-day and hospital mortality rates were 9.0% and 23.2%, respectively. Of 16 patients who died during the hospitalization period, only 1 died of DIC-associated multiple organ failure, while the remaining 15 died of respiratory failure due to AE-fIIP.

Univariate logistic regression analysis revealed that CRP; PaO_2_ /FiO_2_ ratio; and the APACHE II, SOFA, and JAAM-DIC scores were associated with 30-day mortality in AE-fIIP (Table 4). Stepwise multivariate analysis showed that only high DIC score on admission (OR 2.57, 95% CI 1.50–4.40, *P* = 0.0006) was an independent predictor of poor survival (Table 5). A second univariate logistic regression analysis showed that CRP; WBC; platelet count; FDP; D-dimer; PaO_2_ /FiO_2_ ratio; and the APACHE II, SOFA, and JAAM-DIC scores were predictors of hospital mortality (Table 4). Stepwise multivariate logistic regression analysis identified high APACHE II score (OR 1.29, 95% CI 1.01–1.63, *P* = 0.03) and high JAAM-DIC score (OR 3.47, 95% CI 1.73–6.94, *P* = 0.0004) as independent predictors of poor survival (Table 5). BAL findings were not included in the analysis because it was assumed that bronchoscopy was performed only in patients with mild to moderate exacerbations. When the multivariate analysis was adjusted for age, gender, BMI, and smoking status (never versus ever), the JAAM-DIC score on admission became the only independent predictor for both 30-day mortality (OR 7.09, 95% CI 1.35–37.0; *P* = 0.02) and hospital mortality (OR 4.31, 95% CI 1.45–12.83; *P* = 0.008).

**Table 4:** Results of univariate logistic regression analysis for 30-day and hospital mortality in patients with acute exacerbation of fIIP.

	30-day mortality			Hospital mortality		
Variables	Odds ratio	95% CI	*P* value	Odds ratio	95% CI	*P* value
Age, per year	0.99	0.91–1.09	0.93	1.01	0.95–1.08	0.61
Female, sex	0.73	0.13–3.86	0.71	0.46	0.12–1.76	0.25
Classification of IIP	0.25	0.04–1.31	0.1	0.44	0.14–1.35	0.15
FVC	0.62	0.16–2.20	0.47	0.73	0.29–1.84	0.51
FVC, % predicted	0.95	0.91–1.06	0.06	0.97	0.94–1.00	0.13
DLco	1.21	0.71–2.05	0.47	1.13	0.85–1.51	0.38
DLco, % predicted	1.01	0.92–1.10	0.79	1.02	0.98–1.02	0.27
CRP	1.12	1.02–1.23	0.01	1.16	1.07–1.27	0.0004
WBC	1	1.00–1.00	0.3	1	1.00–1.00	0.004
LDH	1	0.99–1.00	0.64	1	0.99–1.00	0.12
Platelet counts	0.91	0.83–1.00	0.06	0.91	0.84–0.98	0.01
Fibrinogen	1	0.99–1.00	0.39	1	0.99–1.00	0.25
FDP	1.03	1.00–1.07	0.05	1.11	1.01–1.22	0.02
D-dimer	1.09	0.98–1.22	0.08	1.13	1.00–1.29	0.04
KL-6	1	0.99–1.00	0.33	1	0.99–1.00	0.18
PaO_2_ /FiO_2_	0.98	0.98–0.99	0.01	0.98	0.97–0.99	<0.0001
APACHE II score	1.24	1.05–1.47	0.01	1.43	1.14–1.80	0.002
SOFA score	1.57	1.06–2.34	0.02	1.93	1.26–2.94	0.002
JAAM-DIC score	2.57	1.50–4.40	0.0006	4.16	2.07–8.40	<0.0001

APACHE II = the acute physiology and chronic health evaluation II, CI = confidence interval, CRP = C reactive protein, DLco = diffusing capacity for carbon monoxide, FDP = fibrinogen and fibrin degradation products, fIIP = fibrosing idiopathic interstitial pneumonia, FVC = forced vital capacity, IIP = idiopathic interstitial pneumonia, JAAM-DIC = Japanese Association for Acute Medicine- disseminated intravascular coagulation, KL-6 = Krebs von der Lungen-6, LDH = lactate dehydrogenase, OR = odds ratio, PaO_2_/FiO_2_ = partial pressure of arterial oxygen / fraction of inspiratory oxygen, SOFA = sequential organ failure assessment, WBC = white blood cell.

**Table 5:** Results of stepwise multivariate logistic regression analysis for 30-day and hospital mortality in patients at first admission for acute exacerbation of fIIP.

	30-day mortality			Hospital mortality		
Variables	Odds ratio	95% CI	*P* value	Odds ratio	95% CI	*P* value
CRP	–	–	–	–	–	–
WBC				–	–	–
Platelet counts				–	–	–
FDP				–	–	–
D-dimer				–	–	–
PaO_2_/FiO_2_ ratio	–	–	–	–	–	–
APACHE II score	–	–	–	1.29	1.01–1.63	0.03
SOFA score	–	–	–	–	–	–
JAAM-DIC score	2.57	1.50–4.40	0.0006	3.47	1.73–6.94	0.0004

A blank cell indicates that the corresponding variable was not incorporated in the analysis. A hyphen indicates that the corresponding variable was incorporated but was not statistically significant.

APACHE II = the acute physiology and chronic health evaluation II, CI = confident interval, CRP = C reactive protein, FDP = fibrinogen and fibrin degradation products, fIIP = fibrosing idiopathic interstitial pneumonia, JAAM-DIC = Japanese Association for Acute Medicine- disseminated intravascular coagulation, OR = odds ratio, PaO_2_ / FiO_2_ = partial pressure of arterial oxygen/fraction of inspiratory oxygen, SOFA = the sequential organ failure assessment, WBC = white blood cell.

Values with significance in the univariate analysis were included in the analysis. WBC, platelet count, FDP D-dimer APACH II score and SOFA score were not included in the 30-day mortality analysis. Only values which were selected as significant are shown.

To reconsider adjustment factors, another multivariate analysis (not stepwise) with variables that were significantly co-related and supposed to be clinically important (JAAM-DIC score, CRP, WBC, and PaO_2_/FiO_2_) was conducted. It also showed that the only JAAM-DIC score (OR 2.35, 95% CI 1.18–4.65, *P* = 0.01) was an independent predictor for 30-day mortality. In the same analysis, the JAAM-DIC score (OR 3.71, 95% CI 1.71–8.06, *P* = 0.0009) and PaO_2_/FiO_2_ (OR 0.98, 95% CI 0.97–0.98, *P* = 0.02) were independent predictors for hospital mortality.

Actual 30-day and hospital mortality in patients with each DIC score are shown in Table 6. As the DIC score increased, the 30-day and hospital mortality rates also increased.

**Table 6:** Thirty-day and hospital mortality according to JAAM-DIC score.

JAAM-DIC score	n	30-day mortality	Hospital mortality
0	49	0 (0)	1 (2.0)
1	17	2 (11.1)*	3 (16.6)*
2≦	15	6 (37.5)*	12 (75.0)*^✝^
Unknown	10	0 (0)	0 (0)

JAAM-DIC = Japanese Association for Acute Medicine- disseminated intravascular coagulation.

Actual numbers are shown, with percentage in parenthesis.

* *P* < 0.05 compared to JAAM-DIC score 0 and

^✝^*P* < 0.05 compared to JAAM-DIC score 1.

Multivariate analysis of the subset of patients who received a definitive diagnosis of IPF identified the JAAM-DIC score on admission as the only independent predictor for both 30-day and hospital mortality (data not shown).

## Discussion

The JAAM-DIC score on admission was significantly associated with both 30-day and hospital mortality in patients with AE- fIIP. To the best of our knowledge, this is the first study to demonstrate the utility of the JAAM-DIC scoring system for predicting survival in AE-fIIP.

Although multiple types of treatments have been evaluated, mortality in patients with AE-IPF remains high [10]. The present study included not only patients with IPF but also patients with all types of fIIP. In this extended patient population, a new indicator, the JAAM-DIC score, was found to predict mortality during acute exacerbation. Furthermore, the DIC score was the strongest predictor of mortality, compared to other surrogate indicators such as PaO_2_/FiO_2_ and other composite indicators such as the APACHE II and SOFA scores.

The JAAM-DIC scoring system was developed to diagnose DIC in critically ill patients [7]. However, the usefulness of the score has been validated in diverse patient populations [7, 16–20]. The scoring system utilizes the SIRS state, platelet counts, PT INR, and FDP. Because of its simplicity and validated usefulness, it is now widely used for diagnosing DIC in Japan.

Coagulation abnormalities in IPF patients have been demonstrated in several reports. The presence of a systemic prothrombotic state has been identified in patients with IPF [23]. Furthermore, elevated levels of serum coagulation factors such as FDP, thrombin-antithrombin complex, plasmin-α2 plasmin inhibitor complex, D-dimer, and thrombomodulin have been reported in patients with AE-IPF compared to patients with stable IPF [24, 25]. Plasma levels of biomarkers of type II alveolar epithelial cell injury and coagulation in patients with AE-IPF are significantly elevated [26]. It was also reported that procoagulant (tissue factor) levels in BAL from IPF patients, particularly those with progressive disease, are higher than in normal patients [27]. These findings suggest that the alveolar luminal compartment in AE-IPF patients may reflect the local parenchymal manifestation of DIC.

DIC contributes to microvascular thrombosis and subsequent multiple organ dysfunction syndrome and is an independent predictor of mortality in critically ill patients [28]. Recently, recombinant human soluble thrombomodulin (rhTM) was developed as a novel anticoagulant treatment for DIC [29]. TM, a glycoprotein expressed on the endothelial cell surface, binds to the thrombin receptor and neutralizes clotting activity. In addition, the thrombin-TM complex increases the activation of protein C [30, 31]. Moreover, rhTM also has anti-inflammatory effects [32]. In Japan, rhTM was approved as a treatment for DIC in 2008. Subsequently, several studies demonstrated mortality benefits of rhTM in patients with DIC [33, 34]. In recent years, it has been reported that rhTM improves survival in patients with AE-IPF and AE-NSIP [6, 21, 35–37]. Therefore, it was speculated that focal, pulmonary DIC might be involved in AE-IPF.

We postulated that the JAAM-DIC score could predict mortality in patients with AE-fIIP and AE-IPF. We found that the 30-day and hospital mortality rates were significantly worse in patients with a DIC score of 1 compared to those with a score of 0, suggesting that a single point is enough to detect an increased mortality risk. The prognosis of patients with more than 2 points was much worse, even though 2 points did not fulfill the criterion for a diagnosis of DIC. A JAAM-DIC score of just 1 or 2 points might indicate a DIC-like reaction in the lung parenchyma, although this supposition remains hypothetical. If the supposition is true, the score would be a useful tool for detecting focal DIC in the lungs of patients with AE-fIIP. In addition to being useful, the JAAM-DIC score is simple to calculate and easy to use. Because of its simplicity, the score can be widely used in clinical practice. Its ability to predict mortality in AE-fIIP was superior to that of the APACHE II and SOFA scores. The ease of use of the JAAM-DIC score is an additional advantage, as the APACHE II and SOFA scoring systems are relatively complex. However, both the JAAM-DIC and the APACHE II scores were identified as independent predictors of hospital mortality by our multivariate analysis. It is possible that these 2 scores evaluate different prognostic aspects of AE-fIIP. The superiority of the JAAM-DIC scoring system may be attributed to the inclusion of SIRS criteria; a recent systematic review and meta-analysis showed that SIRS was superior to the quick SOFA for sepsis diagnosis [38].

This study has several limitations. First, the study was performed at a single center and had a relatively small sample size. Second, the study was conducted in a retrospective fashion. Therefore, the results may not be applicable to different patient populations. Third, the study may have included patients with pulmonary infections. Although we made major efforts to exclude patients with pulmonary infections, clinical and radiological findings of acute exacerbations often resemble those of pneumonia. Fourth, mortality was relatively low (30-day and hospital mortality rates were 8.6% and 17.3%, respectively). A prior study reported a hospital mortality of AE-IPF of 50% [39], although a recent study that included 995 patients with AE-IPF reported higher survival (41% 3-month survival and 44% survival at hospital discharge) [40]. Our comprehensive treatment strategy for AE-fIIP might have improved overall mortality. It is also possible that many patients with NSIP were included in the fIIP group, which would improve survival. However, the 30-day and hospital mortality in patients with diagnosed IPF were also favorable in this study. It is possible that our attempts to diagnose acute exacerbation as early as possible also improved survival. Actually, a favorable hospital survival rate for acute worsening of IPF patients, most of whom were supposed to be with acute exacerbation, of >80% was reported in a recent study [41]. Fifth, the mean lymphocyte count in BAL was high in our study. However, the characteristic BAL findings in patients with AE-IPF and AE-fIIP remain unknown, because no large-scale studies that included BAL analysis have been reported. Actually, we made the diagnosis of AE-fIIP based on a recent international working group report for AE-IPF [10]. The diagnostic criteria for AE-IPF do not include results of BAL analysis. Given that Takei et al. showed that 64.8% of patients with AE-fIIP had a BAL lymphocyte count of >15% [42], some fraction of patients with AE-fIIP have elevated lymphocyte levels in BAL. In the present study, BAL was obtained from 63 patients of 91 with fIIP. It is because patients with severe hypoxemia could not undergo bronchoscopy. If BAL was obtained from all the patients including sever cases, the results may be different. However, further elucidation of BAL findings in additional studies of patients with AE-fIIP is needed.

In conclusion, the JAAM-DIC score on admission was significantly associated with both 30-day and the hospital mortality rates in patients with AE-fIIP. Because of its simplicity of performance and predictive ability for survival, the JAAM-DIC scoring system may be a useful tool for evaluating AE-fIIP severity in general practice.

## Supporting information

S1 Datasheet Baseline data of the study population (n = 91). (XLSX)
